# Nucleation process of the 2011 northern Nagano earthquake from nearby seismic observations

**DOI:** 10.1038/s41598-021-86837-4

**Published:** 2021-04-16

**Authors:** Kengo Shimojo, Bogdan Enescu, Yuji Yagi, Tetsuya Takeda

**Affiliations:** 1 Seismology and Volcanology Division, Japan Meteorological Agency (JMA), Tokyo, Japan; 2 Department of Geophysics, Kyoto University, Kyoto, Japan; 3 National Institute for Earth Physics (NIEP), Magurele, Romania; 4 Faculty of Life and Environmental Sciences, University of Tsukuba, Tsukuba, Japan; 5 National Research Institute for Earth Science and Disaster Resilience (NIED), Tsukuba, Japan

**Keywords:** Seismology, Geophysics

## Abstract

The 2011 magnitude (M) 9.0 Tohoku-oki earthquake was followed by seismicity activation in inland areas throughout Japan. An outstanding case is the M6.2 Northern Nagano earthquake, central Japan, occurred 13-h after the megathrust event, approximately 400 km away from its epicenter. The physical processes relating the occurrence of megathrust earthquakes and subsequent activation of relatively large inland earthquakes are not well understood. Here we use waveform data of a dense local seismic network to reveal with an unprecedented resolution the complex mechanisms leading to the occurrence of the M6.2 earthquake. We show that previously undetected small earthquakes initiated along the Nagano earthquake source fault at relatively short times after the Tohoku-oki megathrust earthquake, and the local seismicity continued intermittently until the occurrence of the M6.2 event, being likely ‘modulated’ by the arrival of surface waves from large, remote aftershocks off-shore Tohoku. About 1-h before the Nagano earthquake, there was an acceleration of micro-seismicity migrating towards its hypocenter. Migration speeds indicate potential localized slow-slip, culminating with the occurrence of the large inland earthquake, with fluids playing a seismicity-activation role at a regional scale.

## Introduction

Huge megathrust earthquakes can cause widespread destruction either because of their violent shaking, or due to their associated natural disasters, like tsunami or induced landslides. Thus, the occurrence of the magnitude (M) 9.0 Tohoku-oki earthquake in 2011 reportedly^1^ caused damage estimated at billions of US dollars and the death of thousands of people. In addition, the accompanying aftershock activity along the plate interface may cause further damage, in particular because such aftershocks could have rather large magnitudes.

Moreover, the 2011 Tohoku-oki earthquake caused widespread activation of seismicity throughout Japanese Islands^2^. Such activity has been induced either by static stress changes (e.g., Toda et al.^3^), dynamic stress changes (e.g., Miyazawa^4^), local excitation of fluids (Shimojo et al.^5^) or a combination of those (e.g., Kato et al.^6^). One of the most remarkable activations of seismicity^5^ took place in northern Nagano Prefecture, central Japan, where a M6.2 crustal earthquake occurred at shallow depth, 13 h after the megathrust event. No seismicity has been detected in the Nagano epicentral area by the Japan Meteorological Agency (JMA) in the 13 h interval; therefore, a causal link between the megathrust event and the occurrence of the relatively large inland earthquake in Nagano region has not been found out.

**Figure 1:**
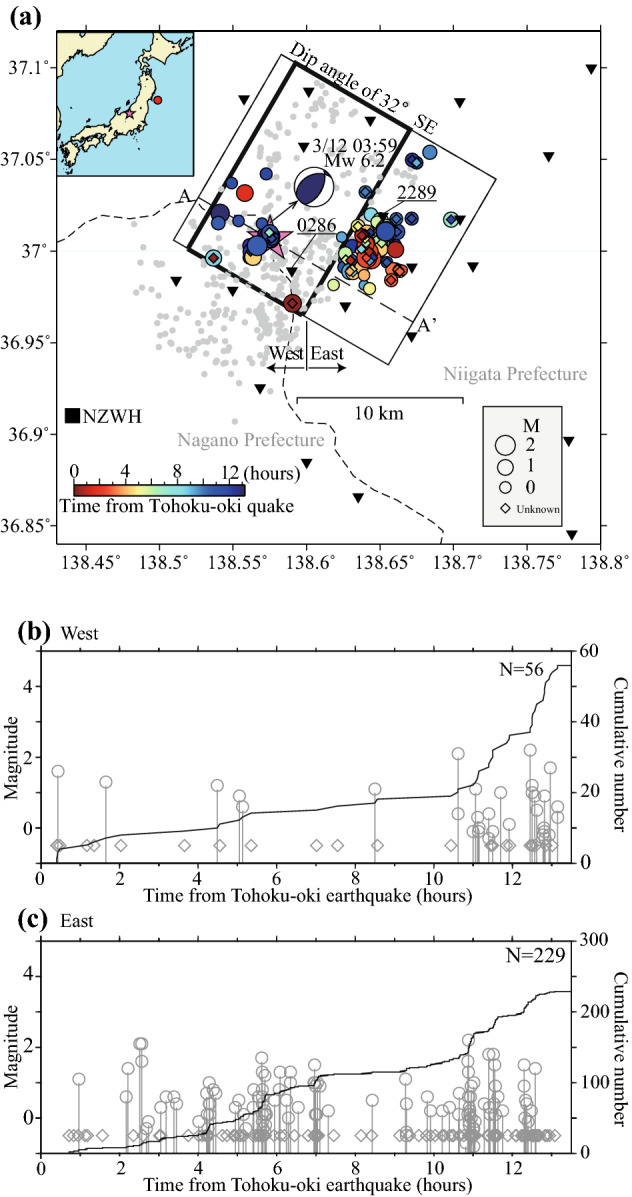
Seismicity distribution in the study region. (**a**) Map view showing the MFT-detected earthquakes. Colored circles and diamonds denote MFT-detections. Size of circles scale with the corresponding events’ magnitude. Diamonds indicate small events for which was not possible to determine reliably magnitudes. Colors indicate lapse time from the Tohoku-oki megathrust event. The one-month relocated Hi-net aftershocks (M ≥ 1.0) are shown in gray. Pink star shows the March 12, 2011 Northern Nagano earthquake. The Moment Tensor focal mechanism of the M6.2 Nagano mainshock, determined by NIED, F-net broadband network is also shown. “West” and “East” indicate the two regions referred in the text. Black thick dashed line shows the profile AA’ in Fig. 2a. The black square and inverted green triangles represent the NZWH Hi-net borehole station and temporary surface stations, respectively. Rectangle with black thick solid line indicates the fault plane^7^ of M6.2 mainshock. The light dashed line shows prefecture border. (**b**,**c**) Time history of seismicity for the “West” and “East” clusters, respectively. Solid gray lines show the cumulative number of events for detections, respectively.

Here we take advantage of the data from a very dense local seismic network (station spacing of 5 km or less—Fig. 1) installed in the Nagano and Niigata regions as early as 2008, allowing to record with an unmatched resolution the seismic/aseismic processes taking place in the area. Based on such data, we reveal for the first time the detailed physical mechanisms leading to the nucleation of the M6.2 earthquake.

### Seismicity in the study area: Initiation of the Northern Nagano sequence by dynamic stresses

The seismic activity in Northern Nagano following the 2011 Tohoku-oki earthquake can be grouped in two distinct regions^5^: the main area where the M6.2 earthquake occurred (Fig. 1a) after about 13 h from the megathrust event, and another one, located 20 km to the south, where a M5.4 earthquake occurred about 1 month later (Figure S1). In our previous study^5^, we detected only 13 events in the main area by applying a matched-filtered technique to the continuous waveforms of 10 borehole stations of the Japanese High Sensitivity Seismograph Network (Hi-net), around the northern Nagano region, using over 1000 events listed in the existing earthquake catalog as template events. In the current research, however, the matched-filtered event detection has been applied to waveform data of a dense local seismic network (Methods section) leading to the detection of 285 micro-earthquakes in the first area. The current earthquake dataset is characterized by a high event completeness (Figure S2), which makes it possible to further divide the seismicity in the first area into an eastern and western cluster (Fig. 1a). The western cluster defines a group of events that occurred in the immediate vicinity of the M6.2 earthquake, while the larger, more voluminous group to the east might belong to a seismologically distinct region.

**Figure 2:**
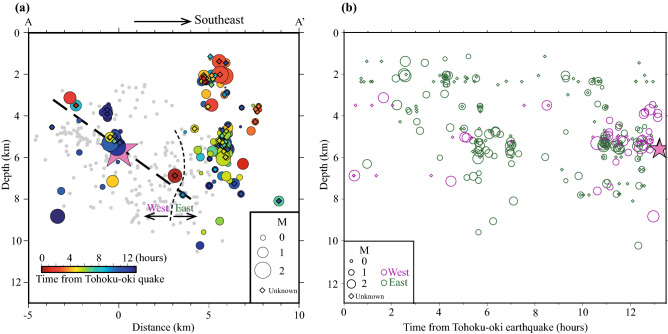
Space–time characteristics of seismicity. (**a**) Cross-section along the profile AA’ in Fig. 1a. “West” and “East” indicate the two regions referred in the text and the curved line shows an approximate separation. Horizontal axis indicates the distance from the M6.2 hypocenter along the AA’ direction. Thick straight dashed line denotes the fault plane^7^ of the M6.2 earthquake. Pink star shows the M6.2 mainshock. Small gray circles indicate one-month relocated Hi-net aftershocks (M ≥ 1.0) of the M6.2 earthquake. (**b**) Depth versus time plot for the MFT-detected events in the study region. Green and blue circles or diamonds correspond to the “East” and “West” clusters, respectively. Size of circles scale with the corresponding events’ magnitude. Diamonds indicate small events for which was not possible to determine reliably magnitudes. Pink star shows M6.2 mainshock.

The earthquake activity in the east was more intense compared to that in the west (Fig. 1) and consisted of 229 events, clustered both in time and space. The eastern cluster is almost vertical, with a discontinuity around 4 km depth (Fig. 2a). As Fig. 2a,b indicate, the earthquake activity in the east started at relatively shallow depths, of about 2 km, and gradually extended towards deeper portions of the seismogenic zone. Moreover, the vertical cross-section (Fig. 2a) suggests the existence of two sub-clusters, a shallower and a deeper one, respectively. The earthquake activity in the west (Fig. 1b) becomes particularly active about 2 h before the M6.2 earthquake, being concentrated close to the hypocenter of the large inland event (Fig. 2a). The hypocenter locations in the west have a SE dipping, which is consistent^7^ with the fault plane of the Nagano mainshock, estimated from aftershock distribution, the focal mechanism of the mainshock and InSAR analysis. Note that waveform inversion analysis indicates that the main rupture of the M6.2 earthquake occurred over an area northeast of its epicenter (roughly coincident with the area delineated by the thick solid rectangle of Fig. 1a)^8^. The aftershocks located off the south side of the rectangle in Fig. 1a may mostly reflect off-fault seismicity activated after the mainshock^7^.

One of the important questions is how and why the seismicity activation in northern Nagano has started. The earliest events of the recorded sequence (Fig. 1) occurred within 30 min. from the origin time of the 2011 Tohoku-oki earthquake, significantly later than the passage of surface waves from the megathrust event, which arrive in northern Nagano just after a few minutes. Nevertheless, considering the seismic sequence in a broader regional context, we propose that the large shaking by the Tohoku-oki surface waves is responsible for the earthquake activation process. Thus, the seismicity in a linear cluster of events about 20 km south of the study region, as well as at a localized spot in the Zigokudani area (Supplementary Information Figure S1), has been activated clearly during the passage of Tohoku-oki surface waves^5^. The activation delay (Fig. 1) could be either genuine, resulting from pore-fluid redistribution^9^ or may reflect some early incompleteness of micro-seismicity (i.e., earthquakes too small to be detected on seismograms, due to their low signal/noise ratio). Since the local dynamic stresses associated with the passage of Tohoku-oki surface waves were relatively large, 300–400 kPa (0.3–0.4 MPa) (Table S3), we infer that they were responsible for initiating the seismicity activation, likely through fluid excitation and/or induced aseismic slip (next sections). The dynamic triggering of seismicity in northern Nagano is further supported by a possible modulation of the locally triggered sequence by the occurrence of larger aftershocks off-shore Tohoku region, as shown in one of the following sections. Note that the static stress changes in the area were either negative or slight positive^5^ (~ 0.02 MPa), thus making them an unlikely mechanism for the activation of seismicity in northern Nagano.

### Enhanced triggering and migration of seismicity in northern Nagano

Following the Tohoku-oki earthquake, northeast Japan experienced negative Coulomb static stress changes, which had as a result an overall decrease of seismic activity^3^, except for several restricted clusters where the relative seismicity increase has been linked to pore fluid pressure changes^10^ and migration of fluids^11^.

**Figure 3:**
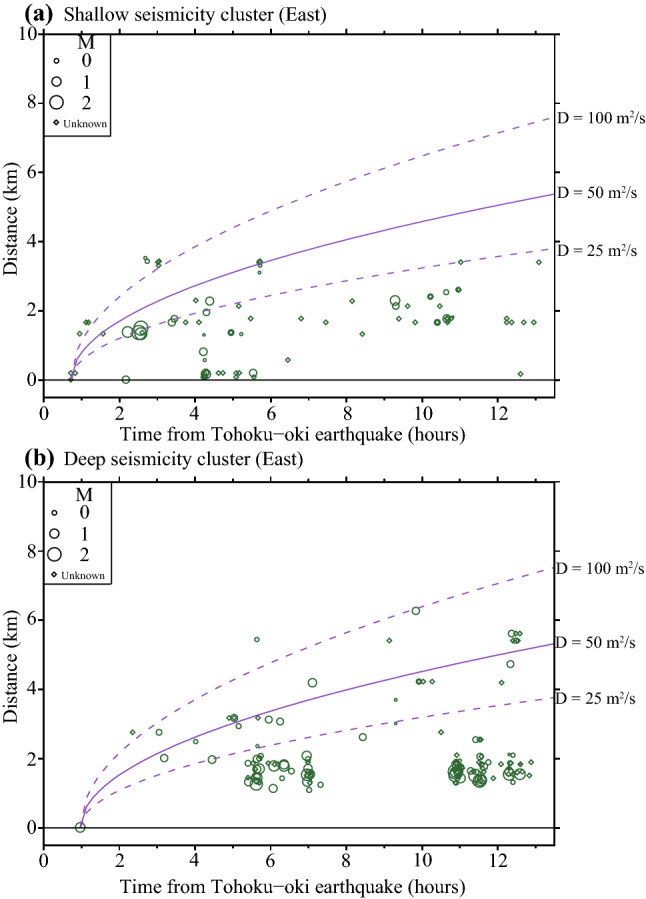
Migration of seismicity within the East cluster. (**a**) Relative distances of events occurred within the shallower sub-cluster versus their occurrence time. (**b**) Same as (**a**) for the deeper sub-cluster. Relative distances are measured relative to the first event of each sub-cluster. Solid purple, upper dashed and lower dashed lines indicate the fluid diffusion front, for a diffusivity parameter, D, of 50, 100 and 25 (m^2^/s), respectively. Size of circles scale with the corresponding events’ magnitude. Diamonds indicate small events for which was not possible to determine reliably magnitudes.

The migration trends of seismicity in the eastern cluster can be visualized in Fig. 3, where the relative event distance from the first earthquake occurring in the shallow sub-cluster (a) and deeper sub-cluster (b) is plotted against time, for each of the events detected during the 13 h period. The two graphs show a clear expansion of the earthquake front with time; if fluid-induced migration is assumed as the underlying triggering mechanism, the distance $r = \sqrt {4\pi Dt}$, from which we obtain a hydraulic diffusivity, D, is in the range 25–100 m^2^/s. Generally, estimated D values for crustal fluids are on the order of 0.01–10 m^2^/s (e.g. Sholtz^12^; Ingebritsen and Manning^13^; Okada et al.^14^). However significantly larger values are also reported in the literature, for example in the case of the Umbria-Marche seismic sequence (22–90 m^2^/s, Antonioli et al.^15^). Note that up-down migration of seismicity as shown in the two sub-clusters has been also documented during shallow earthquake swarms at Yellowstone volcano and interpreted in terms of fluid movement^16^. A detailed tomography study in the area^7^ revealed a relatively large velocity ratio of compressional and shearing waves (*v*_*p*_*/v*_*s*_) at shallow depths, which is an indicator for the presence of fluids (Figure S6 indicates increased *v*_*p*_*/v*_*s*_ in the region of the eastern cluster). The seismicity within the eastern cluster (Fig. 1c) is relatively abundant and the largest events occur within the sequence (not at its beginning), suggesting swarm-like activity, which is often related to the presence of fluids^12^. The reverse fault in this area is steeply dipping to the north and the fracture zone may play the role of fluids’ path for the eastern cluster (see Text S3). The eastern fault zone is likely located in the hanging-wall of the fault plane on which the M6.2 earthquake occurred. Each point from where diffusion starts (Fig. 3), for both the shallow and the deeper cluster, could be assumed as a source of downward pore-pressure diffusion. Fluids at shallow depth flowing towards deeper zones are conceivable, assuming that fluids flow due to differences in the hydrostatic pressure. Considering that the high *v*_*p*_*/v*_*s*_ region is widely distributed throughout the eastern cluster, we hypothesize that fluid diffusion originates from sources at different depths. At shallow depths, fluids are relatively easy to flow along cracks because the permeability of the Earth’s crust decreases with depth. Multiple fluid sources confined at such shallow depths may begin to diffuse to the surrounding areas before those confined in the deeper parts. In this case, the net value of D would be smaller than that estimated above. The initiation of fluid flow may be caused by the dynamic stress changes due to the passage of the surface waves from the Tohoku-oki mainshock-aftershock sequence or the tensional crustal regime induced by the Tohoku-oki earthquake. Thus, in many areas of the Japanese islands, the continental crust in compressional stress regimes was strongly pulled eastward following the Tohoku-oki earthquake^17^. Under such static coseismic extension within the continental crust, channels in the crust may open, allowing fluid flow, especially at shallow depths^18^. On the other hand, the amplitudes of the surface waves from remote large earthquake decrease depending on the depth. Therefore, at shallow locations, the dynamic stress changes by such surface waves are relatively large, and it is expected that the fluid flow due to permeability enhancement may start relatively early. Detailed discussion of the fluid flow initiated by dynamic stress changes will be presented in a later section. Based on the above considerations, we suggest that the enhanced activation of seismicity within the eastern cluster and its gradual spreading towards deeper portions of the seismogenic zone is related to fluid excitation and apparent downward diffusion.

**Figure 4:**
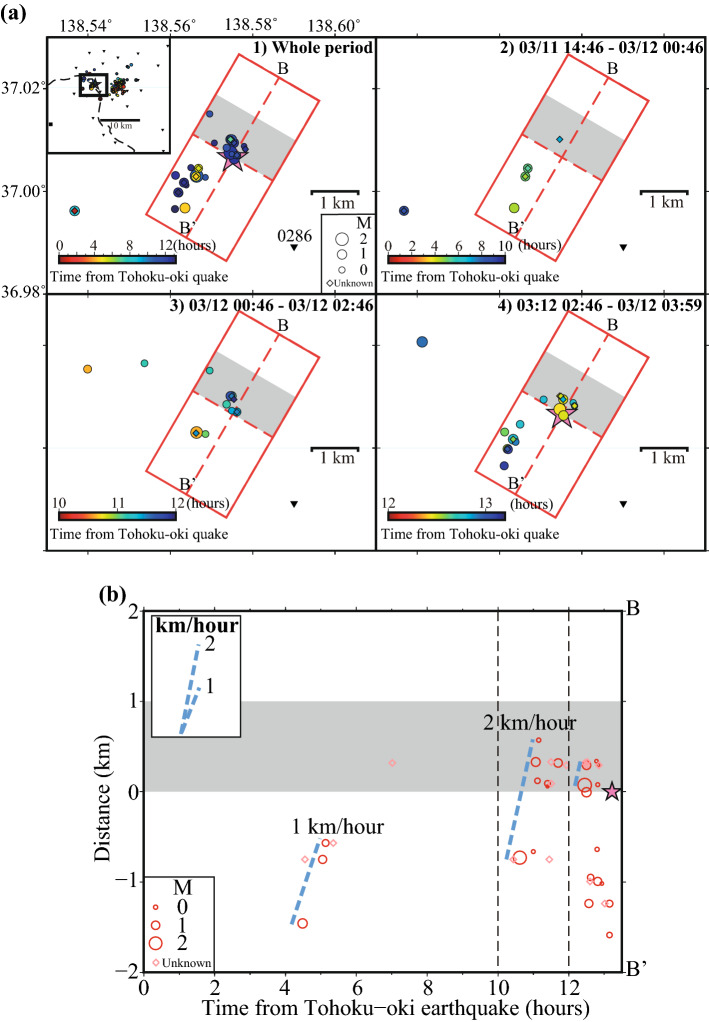
Space and time plots of seismicity for the “West” cluster. (**a**) Maps of detected events for different time windows. Colors indicate lapse time from the Tohoku-oki megathrust event. Pink star shows the M6.2 Northern Nagano earthquake. Gray rectangles indicate the ranges of 1 km NE along strike from the M6.2 earthquake. (**b**) Distance versus time plot along the fault strike of the M6.2 earthquake. Red open circles show events of the cluster plotted as a function of time and distance from the M6.2 mainshock epicenter. Light-blue dashed lines indicate an approximate earthquake migration direction (a rough estimation of the migration speed is written nearby). Size of circles scale with the corresponding events’ magnitude. Diamonds indicate small events for which was not possible to determine reliably magnitudes.

In the western cluster (Fig. 4), on the other hand, many events locate in the immediate vicinity (within about 2 km) of the M6.2 hypocenter. The seismicity was episodically activated three times, migrating along the strike of the M6.2 earthquake source fault, starting southwest of the hypocenter, during 4–7 h, 10–12 h, and more than 12 h after the M9.0 megathrust event (Fig. 4a). The third activation started within just a few hundred meters from the M6.2 hypocenter. The first seismicity migration started about 1.5 km southwest of the M6.2 epicenter, during the passage of surface waves from a larger Tohoku-oki aftershock; seismicity migrated at a speed of ~ 1 km/h (Fig. 4a(2),b). The second migration started about 0.7 km southwest of the epicenter, near the place where the first migration terminated; the migration speed was ~ 2 km/h (Fig. 4a(3),b). The observed migration speeds are in the range of ~ 1 km/day to ~ 1 km/h estimated for aseismic slip (e.g., Yagi et al.^19^; Kato et al.^20^; Lohman and McGuire^21^). Since the seismicity migration is observed very close to the M6.2 earthquake, it is reasonable to relate it with possible aseismic slip along the fault. On the other hand, diffusivities of D = 25–60 m^2^/s are consistent with the observed migration speeds (see the discussion for the eastern cluster). Since a high *v*_*p*_*/v*_*s*_ ratio is observed in southwestern part of this cluster (Figure S7), fluids might be also involved in the observed activation.

### Modulation of local seismicity by the remote Tohoku-oki mainshock-aftershock sequence

While we infer that dynamic stress changes due to the Tohoku-oki mainshock were likely responsible for the onset of local seismicity in northern Nagano, many large aftershocks occurred after the M9.0 Tohoku-oki megathrust event, raising the question of whether dynamic stress changes caused by large remote aftershocks modulated local seismicity. We therefore searched for Tohoku-oki aftershocks of M_JMA_5.5 or larger that may have increased the number of local events (see Text S1 for details). As a result, we have detected five and ten aftershocks that may have potentially caused additional activation in the western and eastern clusters, respectively. Then, by performing Monte Carlo simulations, we have evaluated that the local activity in the northern Nagano region has been significantly increased following five of these remote Tohoku-oki aftershocks.

**Figure 5:**
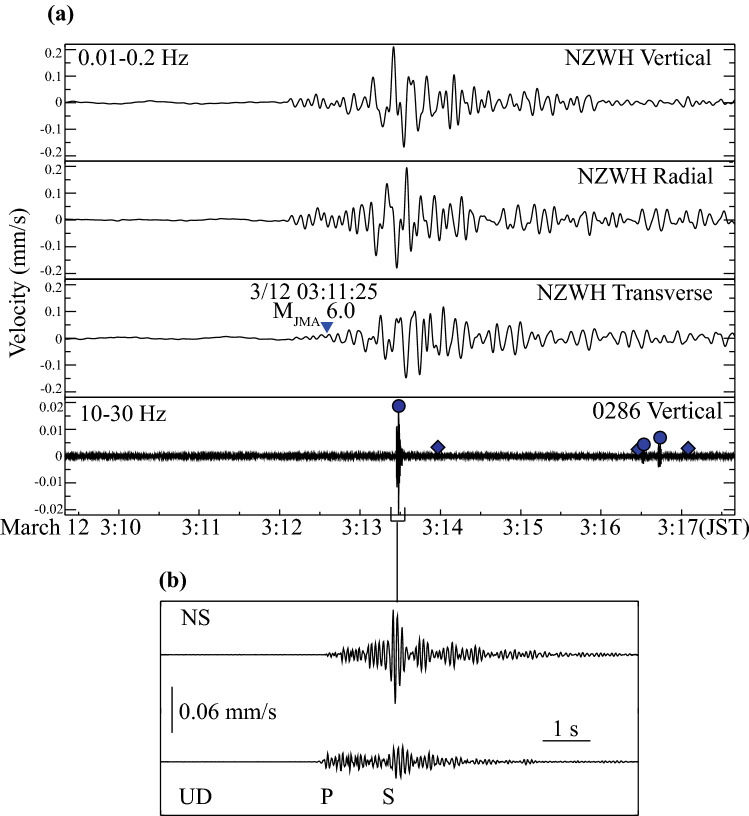
Local earthquakes detected during the passage of surface waves from the M(JMA)6.0 Tohoku-oki aftershock (#7 in Table S3) occurred on March 12, 2011, at 3:11:25 (JST), off-shore Fukushima Prefecture. (**a**) From top to bottom: low-frequency seismograms (0.01–0.2 Hz) at the NZWH station (vertical, radial and transverse components) and high-frequency waveform (10–30 Hz) at the temporary station 0286; locally triggered events are marked by small circles and diamonds, colored function of the lapse time from the Tohoku-oki mega-earthquake (same scale as in Fig. 1). Blue inverted triangles indicate the arrival of Love waves from the Tohoku-oki aftershock, for a phase velocity of 4.1 km/s. (**b**) Enlarged high-frequency seismogram showing P- and S-wave arrivals from the largest event (M2.2, occurred on March 12, 2011, at 3:13:25 (JST)) among those triggered in the “West”.

In both western and eastern clusters, the first local events occurred during the passage of the surface waves from some larger aftershocks (Figure S2 and Table S1). The first and third activation periods within the western cluster initiated immediately after Tohoku-oki aftershocks. Figure 5 shows the third activation period in the western cluster during the passage of surface waves from the M6.0 Tohoku-oki aftershock, which occurred offshore Fukushima Prefecture (03:11:25 JST 12 March 2011). An M2.2 local event, the largest in both the western and eastern clusters, occurred about 50 s after the Love-wave arrival from the M6.0 event and was followed by smaller events in the western cluster. The supplement discusses in detail the significance of triggering in Northern Nagano by these remote Tohoku-oki aftershocks.

### Triggering scenarios for the seismicity activation in northern Nagano: the nucleation of the M6.2 2011 Northern Nagano earthquake

By using waveform data recorded at the NZWH Hi-net station, we estimated the maximum dynamic stresses for seven Tohoku-oki aftershocks that increased the local seismicity significantly or were followed by remarkable activity (Table S3). These values are similar to the minimum dynamic stresses that have been shown to trigger earthquakes in geothermal regions (on the order of 1–10 kPa; e.g., Aiken and Peng^22^). Hence, despite the lack of observations of instantaneous triggering by the Tohoku-oki mainshock, some of its moderate or large aftershocks might have contributed to the activation of seismicity in both the eastern and western clusters. There are two triggering scenarios that may explain the onset and evolution of seismicity due to dynamic stress changes from events of-shore Tohoku.

In the first scenario, distant seismic waves dislodge sedimentary particles blocking flow paths^23^, fracture impermeable media^24,25^, or excite fluids during the dilatational phase of surface waves^26^, leading to seismicity activation. Fluid flow might have been encouraged by repeated shaking due to the passage of surface waves from the M9 event and its subsequent aftershocks. Even though the probability of initiating fluid flow per one passage of surface waves might be relatively low, multiple passages in a relatively short time may rapidly increase this probability.

In the second scenario, transient seismic wavetrains from the events off-shore Tohoku renewed the frictional state of faults^27^ in Nagano, likely through partial damage of frictional contacts^28^. Changes of the rate-and-state^29^ variables by dynamic stress changes on a fault which is close to failure may have caused considerable aseismic slip acceleration^30^, which in turn triggered micro-seismicity.

Note that the fluid flow scenario may involve aseismic slip. Indeed, as recent research suggests, fluid intrusion into a fault with heterogeneous frictional properties may induce aseismic slip and secondary seismicity^31^. Thus, the seismicity in the western cluster might be the byproduct of aseismic slip induced by fluid flow from the area located southwest from the M6.2 fault and accelerated by additional damage of frictional contacts caused by the dynamic stress changes from the Tohoku-oki aftershocks. On the other hand, the third intense activation in the vicinity of the M6.2 hypocenter might be also interpreted as cascade triggering through stress transfer between small foreshocks located close to each other (e.g., Yao et al.^32^; Durand et al.^33^). The culmination of these aseismic processes and/or cascade triggering, within the western cluster, resulted in the triggering of the M6.2 Northern Nagano earthquake.

## Conclusions

We have clarified the seismicity patterns before the occurrence of the M6.2 Northern Nagano earthquake and proposed detailed scenarios to explain the nucleation of the large inland earthquake, as well its relationship with the Tohoku-oki megathrust event.

Our results indicate that the seismicity in the vicinity of the M6.2 Nagano earthquake was likely activated dynamically by the surface waves of both the Tohoku-oki earthquake and its largest aftershocks. Although the dynamic stresses induced by each of the remote aftershocks were relatively small, their repeated shaking modulated the local seismicity activation in northern Nagano, either by facilitating fluid flow, changing the faults’ frictional parameters, or a combination of these factors. Repeated migrations of micro-seismicity in the vicinity of the mainshock hypocenter indicate localized slow-slip, with fluids playing a facilitating role. The final acceleration of seismicity (foreshock activity) that has started about one hour before the Northern Nagano earthquake migrated towards the mainshock hypocenter leading to the occurrence of the large inland earthquake.

Our findings suggest that the detailed monitoring and modeling of seismicity may provide important clues for the forecasting of large inland earthquakes. The detailed investigation of different geophysical processes, like migration of micro-seismicity, swarm-like seismic activity, remote triggering, localized slow-slip and the presence of crustal fluids may lead to successful short-term forecasting of large inland earthquakes. Moreover, understanding the occurrence mechanism of large triggered earthquakes may help estimate the seismic hazard after huge megathrust events.

## Methods

### Earthquake location

We picked the P- and S-wave arrivals of earthquakes on continuous seismograms recorded at the local network seismic stations as well as the NIED seismic station (NZWH) in the region (Fig. 1a), and located the events using the WIN waveform data processing system^34^. Using WIN system’s picking program, we first detect some events to use as first templates for event detection by MFT. Then we obtained 43 first template events at the vicinity of 0286 and 2289 stations (see next paragraph).

### Matched filter technique (MFT) for event detection

The located events were used as the first MFT templates in the study region (Fig. 1a, black thin rectangle). We have applied a two-way 10–30 Hz Butterworth filter to the continuous data from two high-density local network stations, 0286 and 2289 (Fig. 1a), to reduce the influence of the low frequency coda wave of the Tohoku-oki mainshock and aftershocks. We applied the MFT to data from the two stations, following the procedure of Peng and Zhao^35^. The waveforms of all newly identified events were visually checked to minimize the possibility of spurious detection. The events thus detected were relocated by using P- and S-wave arrivals manually picked on seismograms recorded by at least three nearby seismic stations and were then used as new templates. We also applied MFT to the continuous waveform data recorded from 4 h before the Tohoku-oki earthquake until its occurrence, and confirmed that no event was detected in the Nagano region.

### Earthquake relocation

The earthquakes detected by the above procedure were relocated using the tomoDD software^36^ and the 3D-velocity model in the Northern Nagano region^7^. We end up with a catalogue of 285 events (hypocentral errors on the order of tens of meters to several hundreds of meters) for the 13-h time period following the Tohoku-oki mainshock.

### Dynamic stress estimation

Dynamic stresses, σ_d_ were estimated as Gu’/v_ph_ (Jaeger and Cook^37^), where G is the shear modulus (30 GPa), v_ph_ is the phase velocity (considered here as 3.5 km/s for Rayleigh waves and 4.1 km/s for Love waves^38^), and u’ is the peak particle velocity, determined directly from seismograms.

### Magnitudes

The magnitudes of all the earthquakes detected in this study are body-wave magnitudes, determined from the maximum amplitudes of vertical-component seismograms recorded by the dense local seismic network and the NZWH (NIED) station; these magnitudes are in general consistent with the JMA magnitude scale. The magnitude (M6.2) of the Northern Nagano mainshock is a moment-magnitude (Mw), obtained by moment tensor analysis of the F-net (NIED) broadband seismograph network data. All the other event magnitudes are those reported in the JMA earthquake catalog.

## Supplementary Information

Supplementary Information
