# Supplementary material for: Nucleation process of the 2011 northern Nagano earthquake from nearby seismic observations
Source: Sci Rep. 2021 Apr 16;11:8143. doi: 10.1038/s41598-021-86837-4 (PMC8052401; doi:10.1038/s41598-021-86837-4)
Supplement: Supplementary file 1 — Supplementary Information [file 41598_2021_86837_MOESM1_ESM.pdf]

Nucleation process of the 2011 northern Nagano earthquake from nearby seismic observations

*Kengo Shimojo (1), *Bogdan Enescu (2, 3), Yuji Yagi (4) and Tetsuya Takeda (5)

(1) Seismology and Volcanology Division, Japan Meteorological Agency, Tokyo, Japan, shimojo@met.kishou.go.jp

(2) Department of Geophysics, Kyoto University, Kyoto, Japan, benescu@kugi.kyoto-u.ac.jp

(3) National Institute for Earth Physics (NIEP), Magurele, Romania

(4) Faculty of Life and Environmental Sciences, University of Tsukuba, Tsukuba, Japan

(5) National Research Institute for Earth Science and Disaster Resilience, Tsukuba, Japan

Text S1

[Detection of Tohoku-oki aftershocks that could dynamically trigger local seismicity]

Many large aftershocks occurred immediately after the M9.0 Tohoku-oki megathrust event, raising the question whether dynamic stress changes caused by the large aftershocks modulated local seismicity at places located at teleseismic distances from the megathrust's epicenter. Therefore, we have searched for Tohoku-oki aftershocks that possibly triggered local events in the northern Nagano region, using the procedure outlined below.

To identify aftershocks to include in this analysis, we first estimated a threshold magnitude for an event that could potentially trigger local seismicity by dynamic stresses. Van der Elst and Brodsky (2010), using a statistical approach, estimated that the minimum dynamic stresses that could trigger earthquakes are on the order of 0.1 kPa. The peak dynamic stress σ_d is proportional to Gu'/v_{ph} (Jaeger and Cook, 1979), where G is the shear modulus (30 GPa), v_{ph} is the phase velocity (considered here as 3.5 km/s for Rayleigh waves and 4.1 km/s for Love waves, e.g., Peng et al., 2009, Shimojo et al., 2014), and u' is the peak particle velocity (determined directly from seismograms). Thus, dynamic stress changes on the order of 0.1 kPa correspond to peak velocity amplitudes on the order of a few 10^4 of nm/s. A visual check of the low-frequency envelope of the vertical component waveform at station NZWH showed that most peak

amplitudes excited by events larger than M_{JMA}5.5 that occurred in the Tohoku-oki aftershock area (34.38° – 40.11°N, 137.58° – 144.79°E) exceeded a few 10^4 nm/s. Hence, we considered 113 aftershocks of $M_{JMA} \geq 5.5$, which occurred during a 13-hour period after the Tohoku-oki mainshock, as potentially being able to trigger earthquakes in northern Nagano.

To detect remote aftershocks that triggered local seismicity, we first calculated the Love-wave arrival times for the 113 $M_{JMA} \geq 5.5$ Tohoku-oki aftershocks (referred to as large events) in each of the following northern Nagano areas: the eastern and western clusters referred in this paper, as well as the South and Zigokudani areas (Figure S1), where triggered seismicity has been detected by Shimojo et al., (2014) through MFT analysis, using only Hi-net stations. We used the center of the area studied in this paper (37.0226°N, 138.6193°E - the center of the black rectangle in Figure 1) as a reference point for the seismicity in the western and eastern clusters and assumed a Love-wave phase velocity of 4.1 km/s. East of the reference point, where the surface waves of the Tohoku-oki aftershocks would have arrived earlier, there is a possibility that events were triggered before the arrival of surface waves at the reference point. Therefore, we calculated the arrival time differences between the Love-wave arrivals at the reference point and the easternmost point in the research area, shown as a solid rectangle in Figure 1 (i.e., the point expected to show the largest Love-wave arrival time difference). Then, considering such maximum time lags of Love-wave arrivals, the theoretical arrival times are further

shifted by -2.42s. We applied the same procedure for the estimation of the arrival times in the South and Zigokudani areas by using reference points located at (36.8°N, 138.6°E) and (36.776°N, 138.494°E), respectively.

Next, we searched and counted the numbers of local events in windows of 100- and 200-s length, before and after each Love-wave arrival time. To check for potential triggering, we have considered the number of local events identified before (*b*), and after (*a*) these arrival times. For the South and Zigokudani areas, we used the local events detected by Shimojo et al., 2014 (Figure S1).

We then generated 1000 synthetic catalogues by randomly shuffling the time difference between temporally adjacent local events listed in the real catalogue for each area. Then, we have applied the procedure described above to these randomized synthetic catalogues. Since we have used 113 Tohoku-oki aftershocks, there were 113 × 1000 = 113,000 values of a and $a – b$ obtained for each area. We calculated the cumulative frequency distributions of these 113,000 values for a and $a – b$, for each area, and defined the upper 5% of the cumulative distribution as the threshold value for detecting remote aftershocks with “anomalously” large a or $a – b$ values.

Finally, using the thresholds obtained for each area and time window, we have identified the large remote events (i.e., the Tohoku-oki aftershocks) associated either with significant a values (interpreted as remarkable local seismicity) or $a – b$ values (interpreted as significant activation) and considered them as potential triggering events.

We have then visually checked the low-frequency waveforms at station NZWH and excluded from the list of triggering events those earthquakes for which surface wave trains could not be confirmed.

We next calculated *A* and *A* – *B* as the sum of all values of *a* and *a* – *b*, respectively, in each real catalogue of large events ($M_{JMA} \geq 6.0$ or 6.5) to confirm the modulation of the local seismicity in each area through the whole seismicity time series. We also calculated *A* and *A* – *B* for each synthetic catalogue, thus obtaining 1000 *A* and *A* – *B* values for each area. We then calculated the cumulative distributions of those values, and defined thresholds as described above. Values of *A* and *A* – *B* for events larger than $M_{JMA} \geq 6.0$ or 6.5 are compiled in Table S2. In Tables S1 and S2, the threshold values for each event, area, time window, and range of magnitudes are shown at the bottom of each column.

As a result, in both the western and eastern clusters, the first local events occurred during the passage of the surface waves from Tohoku-oki aftershocks (Figure S2 and Table S1). Figure S2 and Table S1 include only moderate or large aftershocks with (a – b) ≥ 1 in either time windows. As examples, seismograms of the first three local events in the western cluster, recorded at station 0286 (Figure 1) during or after the passage of surface waves from an M_{JMA}6.5 event that occurred offshore of Ibaraki Prefecture (15:07:16 JST 11 March 2011) and another M_{JMA}7.4 event that occurred offshore Iwate Prefecture (15:08:53 JST 11 March 2011) are shown in Figure S3. All three events

occurred within the 100 s window of each Love-wave arrival. In the western cluster (Figure 4), the first event of the first and third periods of activation occurred during the passage of surface waves from aftershocks. Figure 5 shows the third activity period during the passage of surface waves from the M_{JMA} 6.0 event that occurred offshore of Fukushima Prefecture (03:11:25 JST 12 March 2011). An M 2.2 event, which was the largest local event in both the western and eastern clusters, occurred about 50 s after the Love-wave arrival from the M_{JMA}6.0 event and was followed by relatively smaller events. We estimate the dynamic stress changes associated with the passage of Love and Rayleigh waves in the area from the amplitudes of the surface wave ground velocities (e.g., Peng et al., 2009). Table S3 presents the estimated maximum dynamic stress changes for seven Tohoku-oki aftershocks that were followed either by a significant increase of local seismicity or a remarkable, increased local activity. The surface wave arrivals from the M_{JMA} 6.5 and M_{JMA} 7.4 aftershocks, which were followed by the earliest local events in the western cluster, about 5 km south of the M6.2 northern Nagano event, were associated with maximum dynamic stress changes on the order of 10 kPa. Similarly, the arrival of surface wave from the M_{JMA} 7.5 event, which was followed by the two earliest local events in the eastern cluster, was also associated with a maximum dynamic change on the order of 10 kPa. The surface wave arrivals from the M_{JMA}6.8 and M_{JMA}6.1 aftershocks, which were followed by significant levels of local seismicity in the western cluster, were associated with maximum dynamic stress

changes on the order of 10 kPa and 1 kPa, respectively. The surface waves arrivals from both the M_{JMA}6.2 and M_{JMA}6.0 events, which were followed by the first and third periods of activity in the western cluster (Figure 4), respectively, were associated with maximum dynamic stress changes of about 1 kPa.

We also confirmed whether local seismicity in the western, eastern, South, and Zigokudani areas was not only dynamically triggered by individual aftershocks occurred in the Tohoku region, but was also modulated throughout the aftershock sequence. In the western, eastern, and South areas, aftershocks did not significantly increase seismicity in any time window or magnitude range (Table S2). In the Zigokudani area, however, aftershocks with magnitudes larger than M_{JMA}6.0 clearly increased local seismicity significantly in most examined time windows. Shimojo et al. (2014) quantified the degree of geothermal activity in the northern Nagano region by using published fluid temperature and flux data (Geological Survey of Japan, 2009) and compared geothermal characteristics among the North (i.e., the western and eastern clusters), South, and Zigokudani areas. As a result, the South and Zigokudani areas were characterized by relatively high fluid temperatures and fluxes than the North area, indicating the presence of geothermal activity. In particular, the highest fluid temperature (~80 °C) and flux (3000 L/min) were observed in the well closest to the Zigokudani area, where seismicity started during the passage of the surface waves from the Tohoku-oki earthquake. The highly modulated seismicity in the Zigokudani, unlike

other areas, may reflect the ease with which permeability and pore fluid pressure can suddenly increase in the Zigokudani area because of, for example, high fluid temperatures and fluxes.

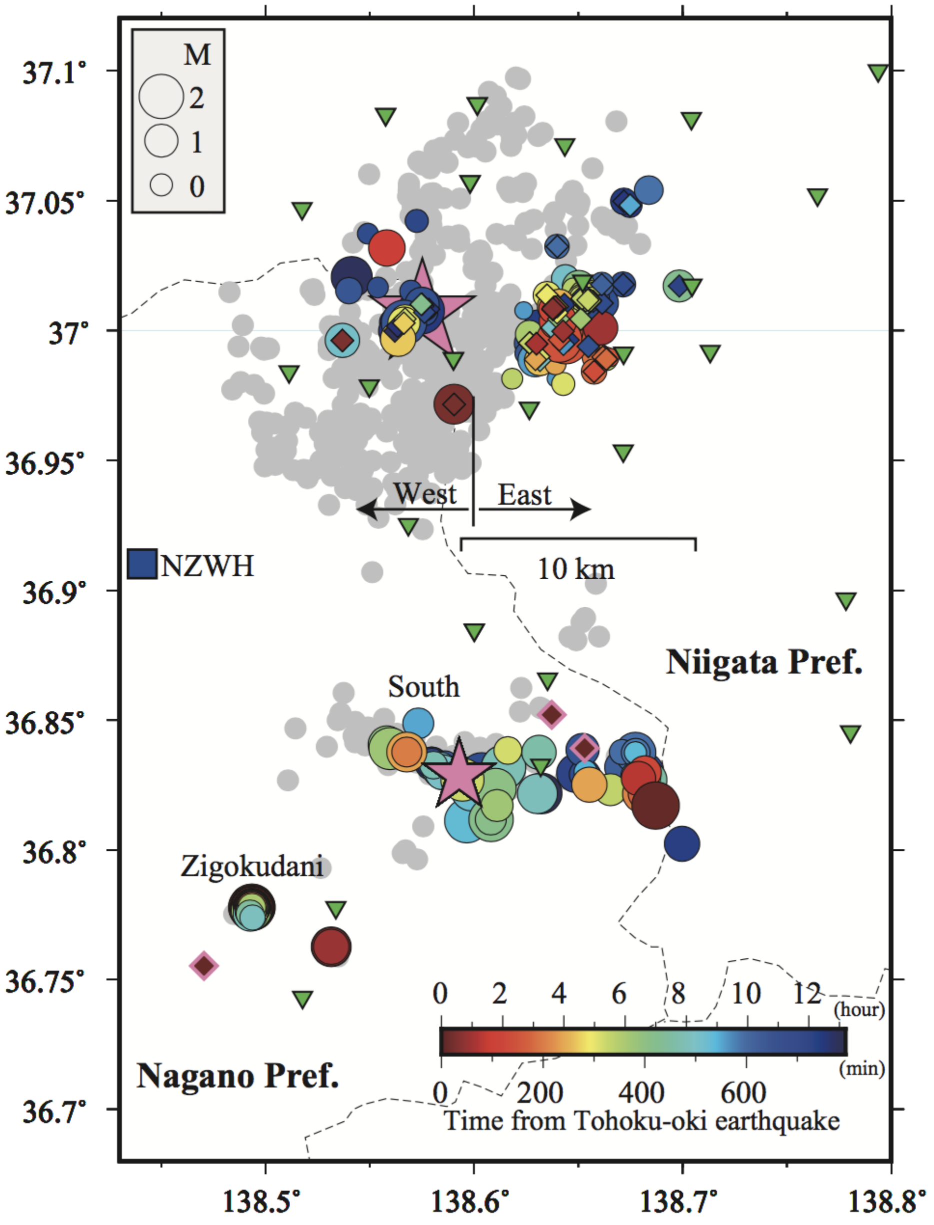

Figure S1. **Earthquakes detected by Shimojo et al. (2014) and in the present study.** The symbols have the same meaning as in Figure 1a. The area "Zigokudani" is a concentrated seismicity spot, characterized by high heat flow, where the occurrence of earthquakes proves to be sensitive to small (remote) stress perturbations.

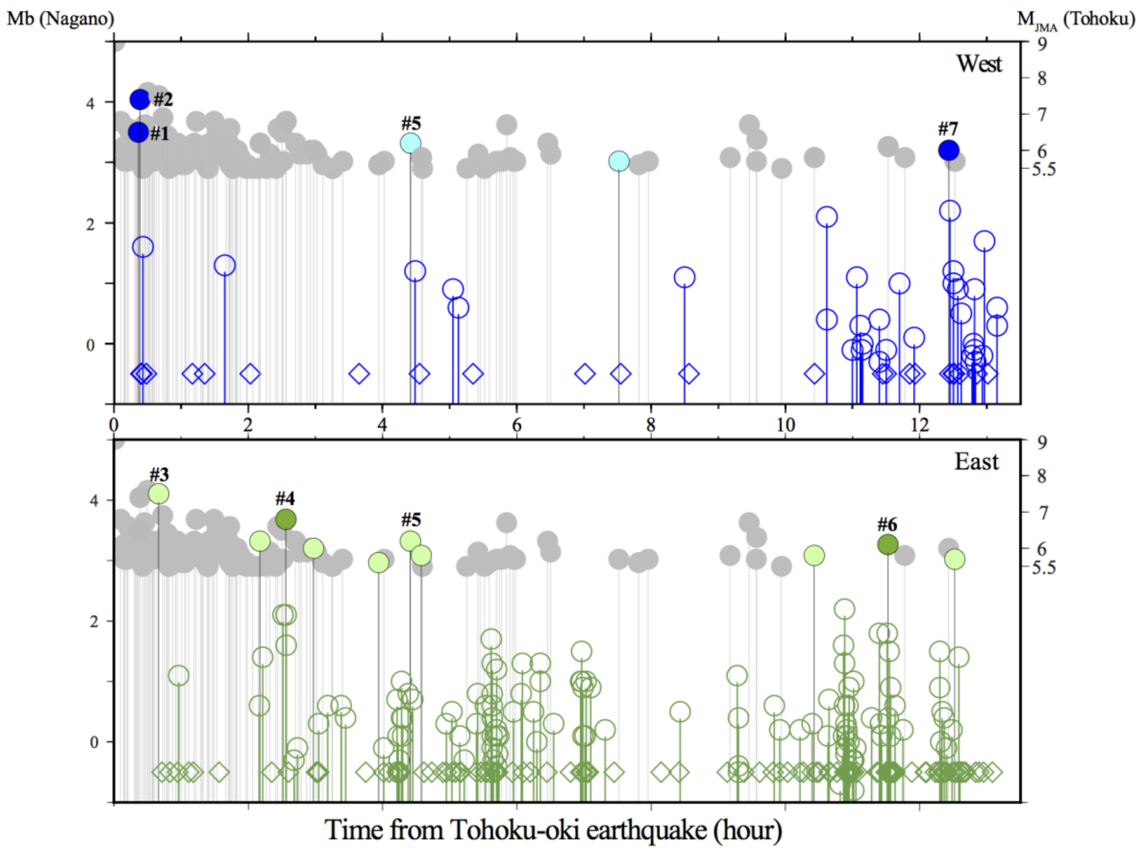

Figure S2. **Time history comparison between the Tohoku-oki sequence (mainshock and large aftershocks) with the local (Nagano) seismicity.** (top) for the "West" cluster. Gray stems indicate Tohoku-oki mainshock and aftershocks (JMA catalog, M ≥ 5.5). Black stems having dark or light blue filled circles show Tohoku-oki aftershocks that are succeeded by a relative increase of local seismicity as compared with the background seismicity. Dark-blue circles are for triggered seismicity that exceeds the statistical significance level. The numbers prefixed by "#" are consistent with the numbers of "remarkable events" of Table S3. (bottom) for the "East" cluster. Symbols have same meaning as in (top), except that blue colors are replaced by green colors.

West

Event information			100s		200s	
Origin time	Location	M_{JMA}	a	a-b	a	a-b
15:07:16	Off shore Ibaraki	6.5	1	1	2	**2**
15:08:53	Off shore Iwate	7.4	**2**	**2**	2	**2**
19:10:39	Off shore Iwate	6.2	0	0	1	1
22:16:48	Off shore Ibaraki	5.7	1	1	1	1
03:11:25	Off shore Fukushima	6.0	**2**	**2**	2	**2**
Threshold			2	2	3	2

East

Event information			100s		200s	
Origin time	Location	M_{JMA}	a	a-b	a	a-b
15:25:16	Off shore Sanriku	7.5	1	1	2	2
16:56:08	Off shore Fukushima	6.2	0	0	1	1
17:19:17	Off shore Ibaraki	6.8	3	**3**	3	2
17:43:52	Off shore Ibaraki	6.0	0	0	2	2
18:42:19	Off shore Sanriku	5.6	0	0	1	1
19:10:39	Off shore Iwate	6.2	1	1	1	0
19:20:24	Off shore Fukushima	5.8	0	0	1	1
01:11:25	Off shore Sanriku	5.8	1	1	1	-1
02:17:25	East Off shore Kanto	6.1	**5**	1	**8**	3
03:17:06	Off shore Ibaraki	5.7	1	0	3	2
Threshold			4	3	5	5

Table S1. **List of M ≥ 5.5 Tohoku-oki aftershocks that have been scrutinized for possible remote triggering in the “West” and “East” Nagano regions, showing statistical test results for their triggering significance.** The tables present some event information (origin time, occurrence cluster and magnitude, as well as “a” and “a-b” values. The letters “b” and “a” denote the number of events, in “100s” and “200s” windows, that occurred before and after the arrival of surface waves; the difference “a-b” is reported in the table. Text in bold indicates numbers that are equal or exceed each of the significance thresholds specified at the bottom, which have been obtained by Monte Carlo simulations.

West

	M_{JMA} ≧6.0 100s		M_{JMA} ≧6.0 200s		M_{JMA} ≧6.5 100s		M_{JMA} ≧6.5 200s	
	A	A-B	A	A-B	A	A-B	A	A-B
Real catalogue	6	4	12	7	3	2	6	6
Threshold	11	8	20	16	7	5	12	9

East

	M_{JMA} ≧6.0 100s		M_{JMA} ≧6.0 200s		M_{JMA} ≧6.5 100s		M_{JMA} ≧6.5 200s	
	A	A-B	A	A-B	A	A-B	A	A-B
Real catalogue	14	4	28	11	4	2	8	5
Threshold	34	18	62	44	18	11	33	22

linear cluster at the South

	M_{JMA} ≧6.0 100s		M_{JMA} ≧6.0 200s		M_{JMA} ≧6.5 100s		M_{JMA} ≧6.5 200s	
	A	A-B	A	A-B	A	A-B	A	A-B
Real catalogue	1	-3	6	0	0	-1	3	1
Threshold	15	10	27	20	9	6	14	10

Zigokudani

	M_{JMA} ≧6.0 100s		M_{JMA} ≧6.0 200s		M_{JMA} ≧6.5 100s		M_{JMA} ≧6.5 200s	
	A	A-B	A	A-B	A	A-B	A	A-B
Real catalogue	**12**	**11**	16	13	**10**	**10**	**12**	**11**
Threshold	11	8	19	15	6	5	11	8

Table S2. **Results of another statistical test to check whether the triggering of Nagano events by larger Tohoku aftershocks is significant.** The tested areas are shown in Figure S1. Besides the "East" and "West" regions, discussed in this paper, we have also tested the triggering for the "South" and the "Zigokudani" areas, both known for geothermal activity. The "A" and "B" symbols represent the sum of individual "a" and "b" (Table S1), respectively, for the time windows (100s and 200s) and Tohoku-oki aftershocks magnitude thresholds (M_{JMA} ≥ 6.0 and M_{JMA} ≥ 6.5, reported in each table header. The numbers in bold indicate statistically significant triggering.

Event information				Maximum dynamic stress changes (kPa)		Significant (or not) (Table S1)	Notes
#	Origin time	Location	M_{JMA}	Love	Rayleigh		
0	14:46:18	Off shore Miyagi	9.0	300	417	-	The M9.0 megathrust event (referred as "mainshock")
1	15:07:16	Off shore Ibaraki	6.5	22	12	Significant	Suceeded by the earliest events in the "West"
2	15:08:53	Off shore Iwate	7.4	31	29	Significant	Suceeded by the earliest events in the "West"
3	15:25:16	Off shore Sanriku	7.5	44	26	Not	Suceeded by the earliest events in the "East"
4	17:19:17	Off shore Ibaraki	6.8	10	12	Significant	
5	19:10:39	Off shore Iwate	6.2	1.4	1.3	Not	Suceeded by first activation in the "West"
6	02:17:25	East off shore Kanto	6.1	1.8	1.6	Significant	
7	03:11:25	Off shore Fukushima	6.0	1.0	1.8	Significant	Suceeded by third activation in the "West"

Table S3. **List of dynamic stress changes associated with the 2011 Tohoku-oki earthquake and several of its aftershocks that have been discussed in the paper.** The columns list the event information (origin time, location and magnitude), the maximum dynamic stresses (kPa) corresponding to the passage of the Love and Rayleigh wave trains, the statistical significance for triggering (Table S1) and some important notes.

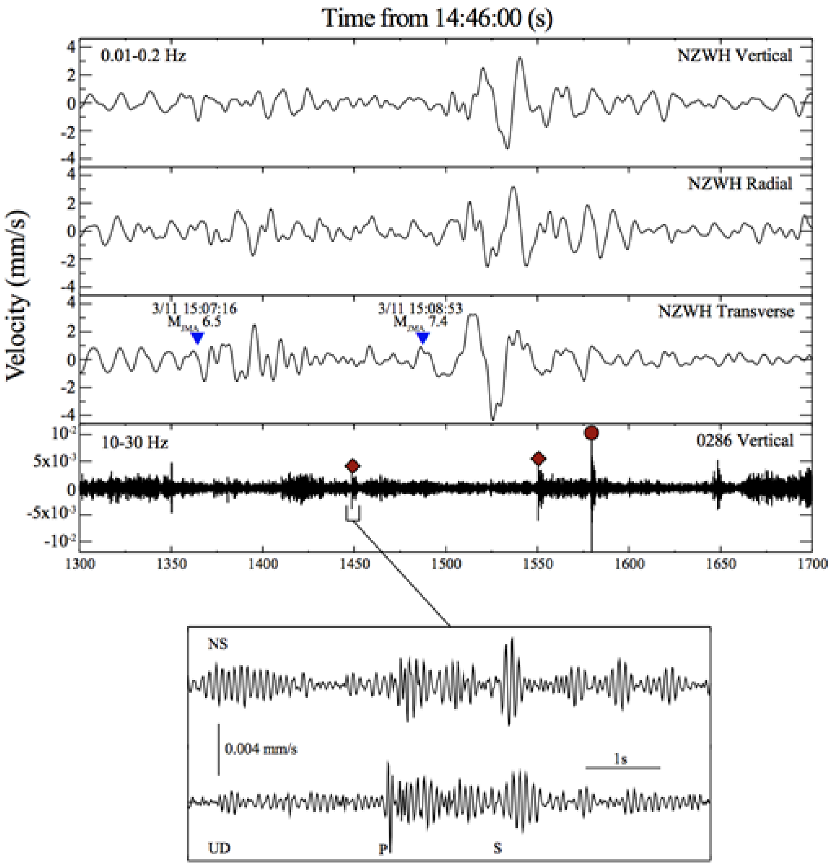

Figure S3. **Earthquakes detected during the passage of surface waves from Tohoku-oki aftershocks.** (top) Top to bottom: low-frequency seismograms (0.01 – 0.2 Hz) at the NZWH station (Vertical, Radial and Transverse components, around the passage of surface waves from the $M_{JMA}7.4$ event (#1 in Figure S2), occurred off-shore Iwate Prefecture (March 11, 2011; 15:08:53, JST)) and high-frequency waveform (10 – 30 Hz) at the temporary station 0286 (Figure 1a); locally triggered events (in the "West" cluster) are marked by small color-filled symbols (same explanations as for the MFT-detections in Figure 1). Blue inverted triangles indicate arrival of Love waves from the M(JMA)6.5 aftershock (#2 in Figure S2), occurred off-shore Ibaraki Prefecture (March 11, 2011; 15:07:16, JST) and the $M_{JMA}7.4$ aftershock, for a phase velocity of 4.1 km/s. (bottom) Enlarged high-frequency seismogram showing P- and S-wave arrivals at 0286 station from one of the triggered events.

Text S2

[Frequency-magnitude distribution of all MFT-detected earthquakes]

In this study, we have detected a total of 285 earthquakes in the source region of the M6.2 Nagano event, compared with only three in the JMA catalogue. The earthquakes are relatively small, with magnitudes less than 2.5. We calculated the magnitude of completeness (Mc) for all events for which magnitudes could be determined, and estimated an Mc of 0.1 (Figure S4), which is significantly less than Mc = 1.2, in our previous study (Shimojo et al., 2014). A total of eight events of the 285 detected events were in the catalogue of the MFT-detected events of Shimojo et al., 2014.

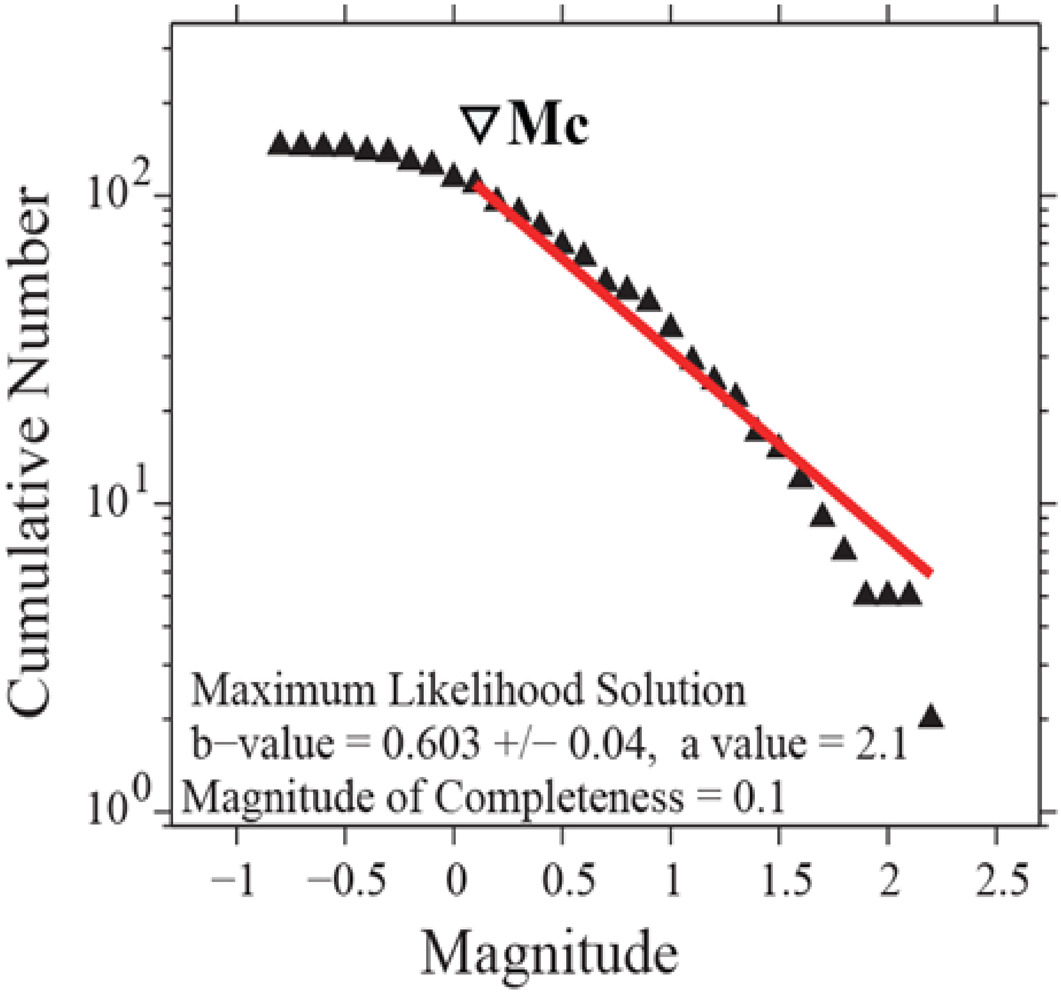

Figure S4. **Frequency-magnitude distribution of all MFT-detected earthquakes in this study.** The black triangles indicate the data, while the Maximum Likelihood fit is shown as a red line. The magnitude of completeness (Mc = 0.1) is computed using the Maximum Curvature method of Wiemer and Wyss (2000). A slightly larger value of Mc (0.4) is obtained if using the Entire Magnitude Range (EMR) method of Woessner and Wiemer (2005).

Text S3

[Fluid migration in the eastern and western clusters]

In the eastern cluster, swarm-like seismicity began in the shallow crust within 1 h of the Tohoku-oki earthquake. Such swarm-like seismicity may indicate that fluid flow caused the pore-fluid pressure to increase (e.g., Scholtz, 2002; Hainzl and Ogata, 2005). The depth of the seismicity in the eastern cluster apparently increased with time from about 2 km to more than 8 km. Fluid diffusion and associated seismicity are often explained by the sudden connection of a relatively high-pressure source to its lower pressure surroundings (Ingebritsen and Manning, 2010), but fluids cannot diffuse from a depth of 2 km to a depth of more than 6 km (as would match the migration of the seismicity in the eastern cluster) because the confining pressure at 2 km depth is comparable to hydrostatic pressure at 6 km depth (assuming a general crustal density of 2.7 Mg/m^3 and gravitational acceleration of 9.8 m/s^2). Figure S5 is a time–distance plot of seismic events from the first event in the eastern cluster. From the time of the earliest event, the fluid diffusion front traces a parabolic curve, corresponding to D = 100–200 m^2/s, where D is the coefficient of diffusion. This diffusivity range is much higher than generally estimated in the crust, even although a few studies have obtained higher values (e.g., Antonioli et al., 2005; Noir et al., 1997). In our research area, the surface structure of the northeast section of the Miyanohara fault, which is a part of the western marginal fault zone of the Tokamachi tectonic basin, indicates a reverse-fault type

displacement with a high dip angle to the north and an east–northeast strike (Ikeda et al., 2002), and the eastern cluster appears to be located from its eastern end, along its extension. A reflection seismic survey conducted in an area about 5 km northeast of concentration area of the eastern cluster, where another surface structure which belongs to the same fault zone as the Miyanohara fault is lying, shows that the north steep dipping reverse fault reaches a depth of about 5 km from the surface (Yokokura et al., 2008). The detailed underground structure in the vicinity of concentration area of the eastern cluster has not been investigated, but the similar fracture zone likely extending to the eastern cluster may play the role of a fluid path. Note that it is considered that the Miyanohara fault is not same as the source fault of M6.2 earthquake since the geometrical features and location of the fault plane are different from those of M6.2 earthquake, as inferred from the source mechanism and the aftershock distribution (Figure1 and 2a); we also assume that the Miyanohara fault is located in the hanging-wall of M6.2 earthquake fault plane with a dipping to the southeast.

On the other hand, there appear to be two sub-clusters of seismicity in the eastern cluster, one at about 2 km depth and the other at about 5 km depth (Figure 2). Figure S6 shows the event depth distribution superposed on the v_p/v_s distribution along the dip direction of the M6.2 event fault. In the eastern cluster, v_p/v_s is high in the seismically active area at around 5 km depth, as well as in the shallower crust. This seismicity distribution may indicate that highly pressurized fluid sources existed close to both

sub-clusters of the eastern earthquake cluster. This model is also expected to satisfy the condition of fluid pressure. In Figures 4, the time–distance distributions from the time of the earliest event in each subcluster are shown for events occurring at depths above and below 4 km. For both subclusters, coefficient of diffusion (D) values were estimated to be on the order of 10 m^2/s; these are more realistic than the range obtained by assuming a single shallow fluid source (Figure S5).

The relatively delayed initiation of activity in the western cluster may also be the result of a fluid-related process. According to tomographic results (Sekiguchi *et al.*, 2013), it is possible that a fluid source exists in or near the southwest part of the cluster where the earthquake migration initiated. Figure S7 shows the depth–distance distribution of the western cluster seismicity along the strike of the M6.2 fault plane superposed on the v_p/v_s distribution. The seismic activity occurred at depths of less than 10 km. On the northeast side of the M6.2 epicenter, the region with high v_p/v_s values is limited to depths shallower than about 3 km, whereas on the southwest side, high v_p/v_s values extend down to about 8 km depth. Thus, the southwest part of the western cluster lies within the region of relatively high v_p/v_s values. The initiation of seismicity in the western cluster may be related to fluids that located near the southwestern high v_p/v_s lobe.

We estimated diffusivity values of ~10 m^2/s for earthquake migration or expansion in both eastern and western clusters. We assumed that the permeability K is proportional to

$D\varepsilon\gamma\ C_T$ (Noir *et al.*, 1997), where D is diffusivity, ε is porosity, γ is viscosity, and C_T is the isothermal compressibility coefficient. Using nominal values $\varepsilon = 0.05$, $\gamma = 2.0\times10^{-4}$ Pa·s, and $C_T = 4.0\times10^{-10}$ Pa^{-1} for water under temperature and pressure conditions of around 5 km depth, we estimated the permeability to be $4.0\times10^{-14} - 4.0\times10^{-13}$ m^2. This permeability is 0–5 orders of magnitude larger than expected at depths of 1–10 km in typical brittle crust (Ingebritsen and Manning, 2002) and is roughly consistent with the relatively high values sometimes associated with earthquake migration or expansion involving the flow of highly pressurized fluids (Ingebritsen and Manning, 2010). We speculate that the relatively high permeability values in this study indicate fluid diffusion from highly pressurized source areas to lower pressure areas, which may have resulted from temporarily enhanced permeability due to the influence of the Tohoku-oki earthquake.

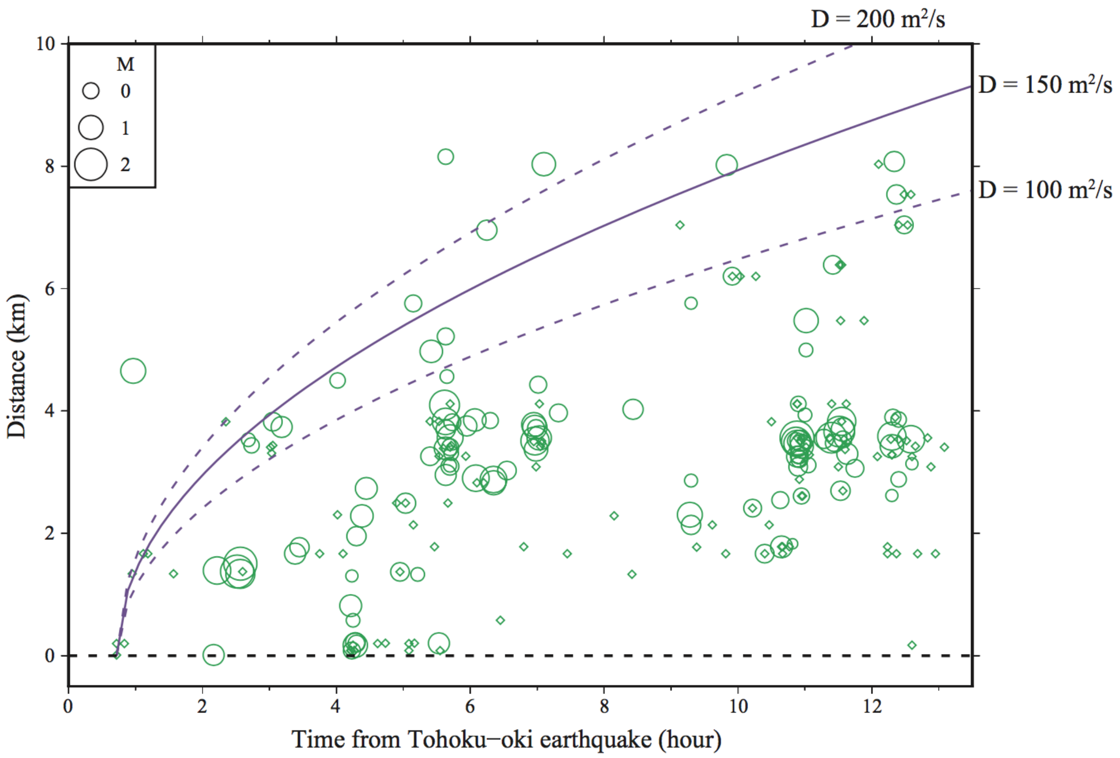

Figure S5 **Distance versus time for the overall “East cluster”.** Distances of events are relative to the first event occurred in the “East” cluster. Solid purple, upper dashed and lower dashed lines indicate the front of fluid diffusion (Shapiro et al., 1997) for a diffusivity, D, of 150, 200 and 100 (m^2/s), respectively.

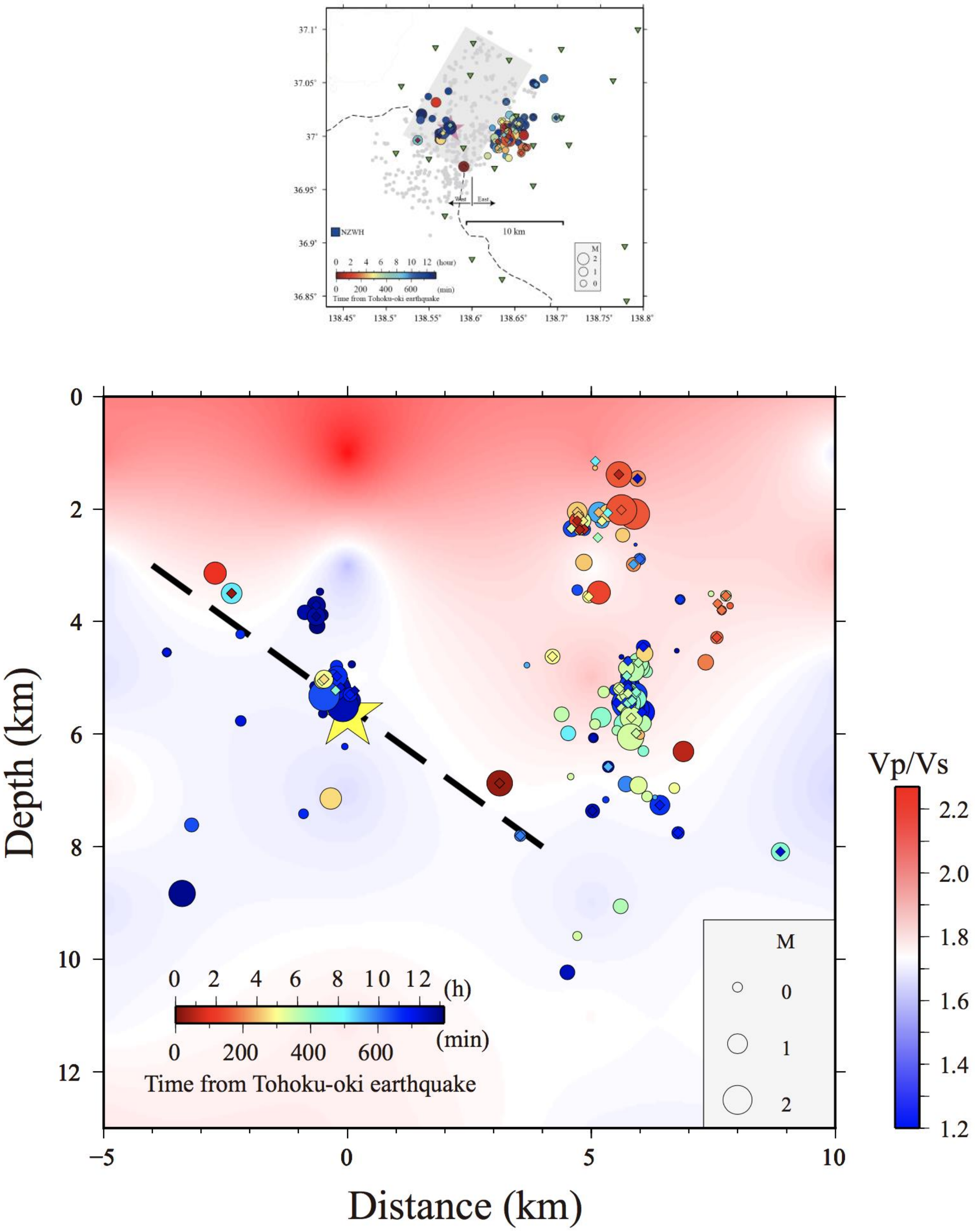

Figure S6. **MFT-detected events overlapped on the Vp/Vs velocity structure.** (top) Same map as in Figure 1(a), with a solid blue line indicating the profile for the cross-section below; (bottom) Cross-section showing the seismicity distribution (same as Figure 2(a)) overlapped on the Vp/Vs structure (Sekiguchi et al., 2013).

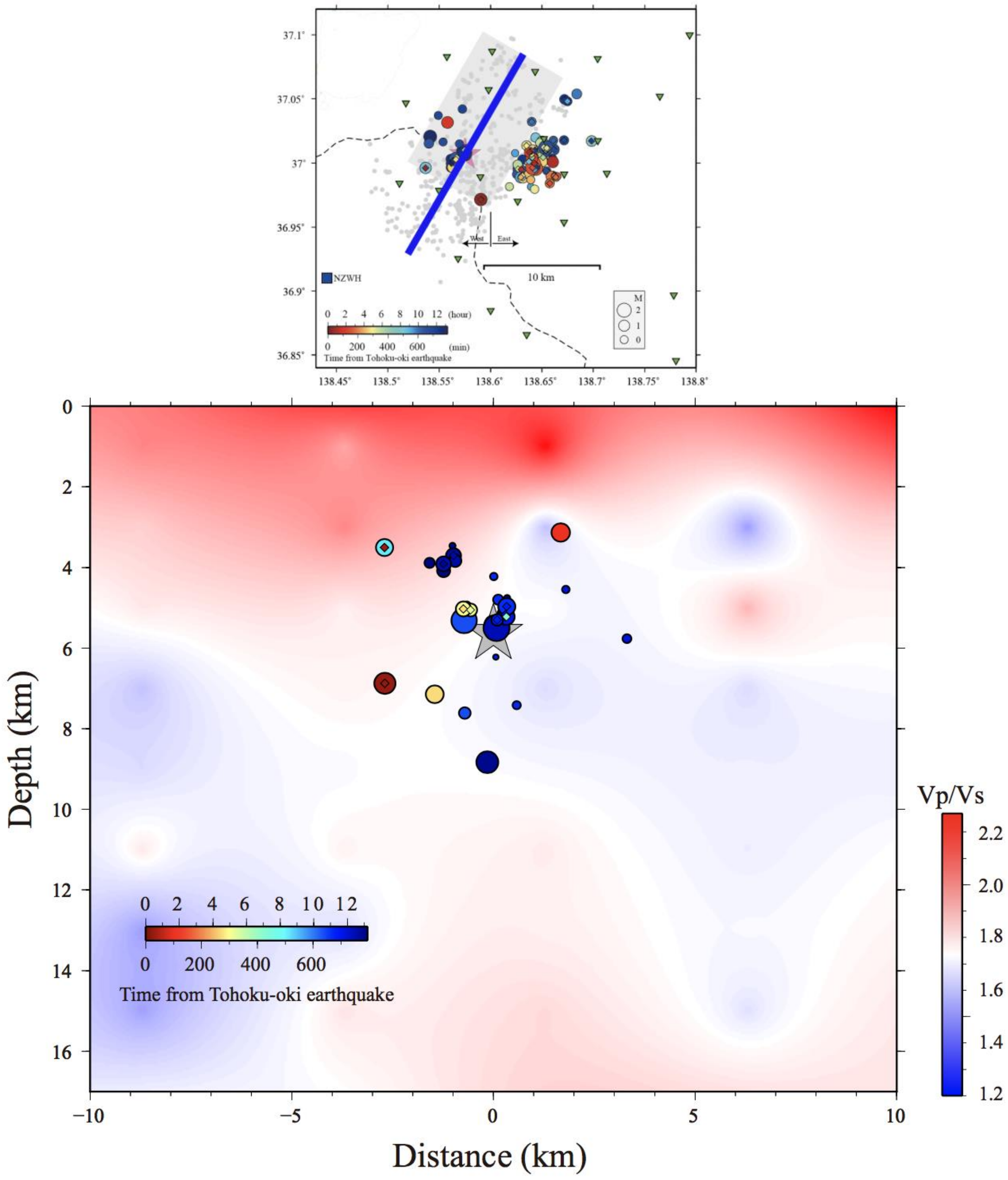

Figure S7. **MFT-detected events (West cluster) overlapped on the Vp/Vs velocity structure.** (top) Same map as in Figure 1(a), with a solid blue line indicating the profile for the cross-section below; (bottom) Cross-section showing the seismicity distribution (West cluster) overlapped on the Vp/Vs structure (Sekiguchi et al., 2013).

References

1) Antonioli, A., Piccinini, D., Chiaraluce, L., & Cocco, M. Fluid flow and seismicity pattern: Evidence from the 1997 Umbria-Marche (central Italy) seismic sequence. *Geophys. Res. Lett.* **32**, L10311 (2005).
2) Geological Survey of Japan, Geothermal potential map of Japan, CD-ROM version. *Digital Geological Map GT-4* (2009).
3) Hainzl, S., & Ogata, Y. Detecting fluid signals in seismicity data through statistical earthquake modeling. *J. Geophys. Res.*, 110, B05S07 (2005).
4) Ingebritsen, S.E. & Manning, C.E. Diffuse fluid flux through orogenic belts: Implications for the world ocean. *Proc. Natl. Acad. Sci. U.S.A.* **99**, 9113-6 (2002).
5) Ingebritsen, S. E. & Manning, C.E. Permeability of the continental crust: dynamic variations inferred from seismicity and metamorphism. *Geofluids* **10**, 193-205 (2010).
6) Jaeger J.C. & Cook N.G.W. Fundamentals of rock mechanics. *3rd Ed. Chap- man and Hall, New York* (1979).
7) Noir, J., Jacques, E., Bekri, S., Adler, P.M., Tapponier, P. & King, G.C.P. Fluid flow triggered migration of events in the 1989 Dobi earthquake sequence of central Afar. *Geophys. Res. Lett.* **24**, 2335–2338 (1997).
8) Peng, Z., Vidale, J.E., Wech, A.G., Nadeau, R.M. & Creager, K.C. Remote triggering of tremor along the San Andreas Fault in central California. *J. Geophys. Res.* **114**, B00A06 (2009).
9) Shimojo, K., Enescu, B., Yagi, Y., & Takeda, T. Fluid-driven seismicity activation in northern Nagano region after the 2011 M9.0 Tohoku-oki earthquake. *Geophys. Res. Lett.* **41**, 7524–7531 (2014).
10) Scholz, C. H., The Mechanics of Earthquakes and Faulting. *Cambridge Univ. Press, New York* (2002).
11) Sekiguchi, S. et al., Onshore earthquake observations, The Annual Report of the Multidisciplinary Research Project for Investigations in the High Strain Rate Zone of Japan. *Research and Development Bureau of MEXT, Japan, and NIED,* 11-49 (2013).
12) Van der Elst, N. J. & E. E. Brodsky, Connecting near-field and far-field earthquake triggering to dynamic strain. *J. Geophys. Res.* **115**, B07311 (2010).

13) Wiemer, S. & Wyss, M. Minimum Magnitude of Completeness in Earthquake Catalogs: Examples from Alaska, the Western United States, and Japan. *Bull. Seismol. Soc. Am.* **90**, 859–869 (2000).
14) Woessner, J. & Wiemer, S. Assessing the quality of earthquake catalogues: Estimating the magnitude of completeness and its uncertainty. *Bull. Seismol. Soc. Am.* **95**, 684–698 (2005).
15) Ikeda, Y., Imaizumi, T., Hirakawa, K., Miyauchi, T. and Sato, H. Atlas of Quaternary Thrust Faults in Japan. *University of Tokyo Press* (2002) (in Japanese).
16) Yokokura et al., Subsurface structure around the Tokamachi fault zone, Niigata prefecture, central Japan, revealed by seismic reflection survey, *Chishitsu News*, **649**, 23-31 (2008) (in Japanese).
